# Macroscopic and Microscopic Properties of Alkali-Activated Slag Recycled Cementitious Material

**DOI:** 10.3390/ma18102212

**Published:** 2025-05-10

**Authors:** Jing Zhu, Zhiming Li, Ying Huang, Yuankai Li

**Affiliations:** 1 College of Civil Engineering and Architecture, Harbin University of Science and Technology, Harbin 150080, China; lizhiming0506@163.com (Z.L.); 13696334828@163.com (Y.L.); 2 Department of Civil Engineering, North Dakota State University (NDSU), Fargo, ND 58102, USA

**Keywords:** alkali-activated slag recycled concrete, recycled material, control variable method, macroeconomic analysis, microscopic analysis

## Abstract

Alkali-activated slag recycled cementitious material (ASRCM) has emerged as a sustainable construction material alternative due to its potential for industrial byproduct valorization and reduced carbon footprint. To study the effect of recycled material content on ASRCM performance, this paper systematically investigates the optimal dosages of recycled stone powder, recycled rubber, and flax fiber in ASRCM with a controlled variable method. The synergistic effects of the inclusion of recycled stone powder, recycled rubber, and flax fiber on macro-microstructural properties on the ASRCM have been analyzed. The results show that the incorporation of recycled materials can significantly enhance both the mechanical properties and workability of the composite, thereby improving the overall stability and performance characteristics of the material system. However, challenges remain in standardizing recycled material reactivity assessment and mitigating long-term durability concerns. More research is needed to investigate the service life and field-scale implementation of ASRCM to accelerate circular economy transitions of the construction sector in the future.

## 1. Introduction

The rapid urbanization process worldwide has brought significant challenges in the management of solid waste and environmental impacts. For instance, in China, urbanization has resulted in the accumulation of approximately 60 billion tons of bulk solid waste, with an annual increase approaching 3 billion tons. The development of sustainable recycling technologies for bulk solid waste and the advancement of green, low-carbon alternative cementitious materials have become critical research priorities in the field of construction materials science, addressing both environmental concerns and resource utilization challenges [1,2,3].

Due to the excellent properties such as high temperature resistance, corrosion resistance, good impermeability, and low energy consumption and emissions, alkali-activated slag recycled cementitious material (ASRCM), as a new type of calcium–aluminosilicate-based inorganic polymer cementitious material, has been considered as a potential replacement for cement. The concept of ASRC was proposed by Davidovits in the 1970s [4]. As a potential substitute for cement, it has received extensive attention in multiple fields [5,6,7,8]. ASRC is produced by using alkali compounds or alkali-containing industrial wastes as activators to activate industrial wastes, supplemented with recycled stone powder, recycled rubber, and flax fiber for modification.

The compressive strength of ASRCM is higher than 50 N/mm^2^, and its bonding performance is basically the same as that of epoxy organic adhesives. Furthermore, its compressive strength after high temperature does not decrease significantly when it experiences a maximum temperature of lower than 600 °C [8]. The composition of ASRCM completely excludes cement, avoiding the traditional cement “two-grinding and one-burning” link. It has the characteristics of a simple production process, low investment, low energy consumption, low pollution, and excellent mechanical properties. It not only has good reduction in noise, dust, CO_2_, and toxic gas but also saves fuel, electricity, and other resources, resulting in significant reduction in environmental pollution [9,10,11].

Among different industrial wastes, flax fiber has high fracture strength and good toughness, without affecting the chemical properties of the material. Under the premise of doping, flax fiber can make the performance of ASRC enhanced. In addition, the flax fiber is degradable and will not pose a threat to the environment [12]. On the other hand, recycled stone powder can improve the mechanical properties of the material, the recycled rubber can improve the frost resistance of the material, and the flax fiber has high fracture strength and good toughness. Without affecting the chemical properties of the material under the premise of doping, flax fiber can make the ductility of ASRCM enhanced, and the fiber itself can be degraded, resulting in no threat to the environment. Finally, recycling materials such as stone powder, recycled rubber, and flax fiber are recycled materials, which significantly reduces raw material procurement costs and waste disposal expenditures while mitigating dependence on natural resources and enhancing resource utilization efficiency at the same time [13,14,15,16,17].

Extensive research has been conducted globally on alkali-activated concrete materials. Reddy et al. [18] activated granular blast furnace slag using NaOH, Na_2_SiO_3_, CaO, Na_2_SO_4_, and MgO, revealing that carbonation induced complete decalcification of C-S-H gel and the formation of highly cross-linked aluminosilicates. Paillard et al. [19] employed NMR, XRD, bound water quantification, and isothermal calorimetry to compare reaction pathways of slag under NaOH, Na_2_CO_3_, and Na_2_Si_1.7_O_4.4_ activators. Their findings demonstrated that NaOH activation promotes the co-formation of well-ordered C-A-S-H gel and hydrotalcite, yielding enhanced early-stage mechanical strength. Zhao [20] synthesized alkali-activated binders from slag and fly ash, observing that mechanical performance improved proportionally with alkali content, while a 7:3 slag-to-fly ash ratio induced maximum drying shrinkage.

However, the existing literature lacks comprehensive studies on systematically investigating the synergistic effects of diversified recycled material incorporations, including recycled stone powder, recycled rubber, and flax fiber, and their optimal dosage ratios on both mechanical performance and workability characteristics of ASRCM. Particularly, the combined effects of material composition ratios on the microstructural evolution and interfacial transition zone formation within ASRCM matrices have not been thoroughly investigated. Thus, there is a research gap necessitating systematic investigations through advanced multiscale characterization techniques to elucidate the intrinsic relationships between composite formulations, hydration mechanisms, and macroscopic engineering properties [21,22,23].

This study addresses such a gap through a controlled variable approach to evaluate the flexural and compressive strength of ASRCM specimens by adjusting the proportions of these recycled components. Macrostructural and microanalytical techniques are combined to identify optimal ASRCM mix designs, elucidate their mechanical behavior and failure mechanisms, and provide theoretical foundations and decision-making support for revising industry standards [24,25,26].

## 2. Material Proportions and Experimental Design

### 2.1. Materials

To improve the workability and mechanical properties of ASRCM for large-scale use, it is necessary to fill the microcracks and pores inside the alkali slag cementitious material. Therefore, in this study, recycled stone powder, recycled rubber, and flax fiber were added to the material, and the dosage of the three was systematically optimized through experiments so that the final material has balanced mechanical properties and drying shrinkage performance. Thus, the materials used in this study include slag, potassium water glass, and flax fibers, and their treatment and mix design are detailed in this section.

#### 2.1.1. Slag

According to the requirements of Granulated Blast Furnace Slag Powder Used in Cement, Mortar and Concrete [27]. The slag was supplied by Liaoyuan Jingang Cement Co., Ltd. (Liaoyuan, China), classified as Grade S95 with a specific surface area of 475 m^2^/kg (Blaine method, ISO 679:2009). Key reactivity indices were quantified as follows:

- Mass coefficient of 1.91;
- alkalinity coefficient of 1.03;
- activity coefficient of 0.44.

**Figure 1:**
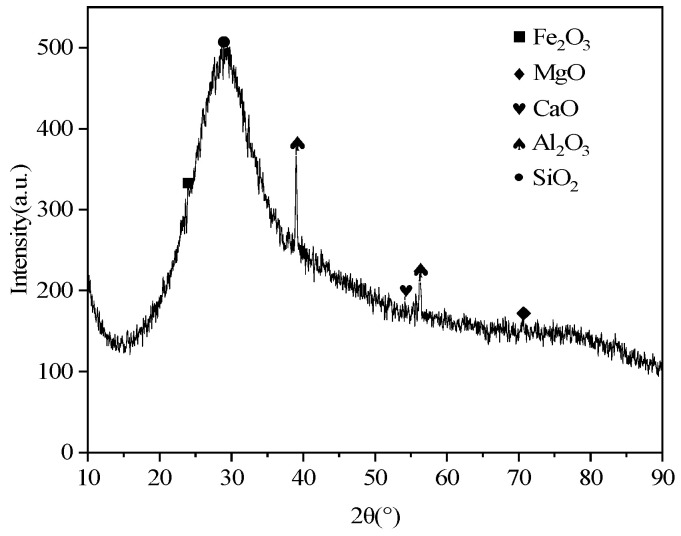
XRD pattern of slag.

The slag’s chemical composition and XRD pattern are shown in Table 1 and Figure 1, respectively. As can be seen in Figure 1, the bread loaf-shaped peak represents amorphous SiO_2_, with almost no distinct characteristic peaks, indicating that the slag primarily exists in an amorphous glassy state.

#### 2.1.2. Potassium Water Glass

The potassium silicate utilized in this study primarily comprises 16.84% potassium oxide (K_2_O) and 26% silicon dioxide (SiO_2_), with a brix value of 39.5, a density of 1.439 g/cm^3^, and a module of 2. It was supplied by Xingtai Dayang Chemical Co., Ltd. (Xingtai, China).

#### 2.1.3. Recycled Stone Powder

Recycled stone powder, provided by Harbin Zhongpeng Building Materials Co., Ltd. (Harbin, China). It exhibited micron-scale particle morphology with a density of 2.53 g/cm^3^, hydrophilicity coefficient of 0.66, and average particle size of 6.6 μm. Its chemical composition is summarized in Table 2.

#### 2.1.4. Recycled Rubber Powder

The recycled rubber powder used in this study has 10 mesh and a particle size of 2 mm. It was sourced from Dujiangyan Huayi Rubber Co., Ltd. (Chengdu, China). It features a specific surface area of 1–50 m^2^/g and a density of 1.0–1.3 g/cm^3^, with high thermal stability, tensile strength, and elasticity.

#### 2.1.5. Flax Fibers

Flax fibers, supplied by Jinan Ruizee Engineering Materials Co., Ltd. (Jinan, China), were used in this study. The density of flax fibers is 1.45 g/cm^3^, with measured morphological dimensions of 36–60 mm in length and 65.00 μm in diameter. The tensile strength is usually from 1200~1500 MPa, with better ductility, abrasion resistance, and resistance to chemicals.

#### 2.1.6. Other Reagents

Sodium hydroxide, NaOH (≥96.0% purity), a strongly alkaline compound, was used in this study, which was provided by Harbin Ligong Chemical Reagent Co., Ltd. (Harbin, China).

The standard sand with SiO_2_ content > 96%, ignition loss ≤ 0.40%, and clay content ≤ 0.20% was applied in this study, and it was sourced from Xiamen ISO Standard Sand Co., Ltd. (Xiamen, China).

The water used in this paper was provided by the Harbin municipal supply and served as the mixing water.

### 2.2. Mix Ratio Design

#### 2.2.1. Pre-Treatment of Flax Fibers

**Figure 2:**
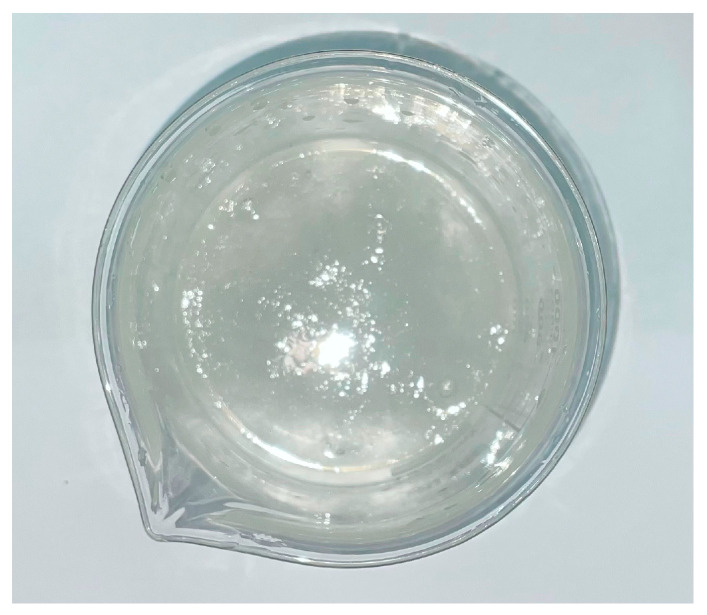
The treatment diagram of flax fiber.

Untreated flax fibers are in the form of cotton felt, and directly adding AASRM and stirring can easily cause uneven fiber distribution. To achieve the optimal combination of flax fiber and AASRM, pretreatment is required. The specific method is to first disperse the flax fiber into small units as much as possible and then add them to an alkaline excitation solution for a period of presoaking. Subsequently, quickly stir with a glass rod to ensure uniform distribution of fibers in the solution, as shown in Figure 2.

#### 2.2.2. Adjusting the Modulus of Potassium Silicate to 1.0

According to the experimental requirements, the optimal modulus of potassium silicate for preparing ASRCM is 1.0. The chemical composition molar ratio of the original potassium water glass is SiO_2_:K_2_O = 2.78:1. To achieve modulus adjustment, NaOH needs to be added to the solution so that the adjusted molar ratio satisfies SiO_2_: (K_2_O + NaOH) =2.78: (1.78 + 1). Each gram of potassium water glass contains 0.155 g of potassium oxide, which is converted to a molar mass of 0.0017 mol. To adjust the modulus to 1.0, the required amount of sodium hydroxide to be added per gram of potassium water glass is 0.0017 mol × 1.78 × 2 = 0.006052 mol (coefficient 2 derived from the equivalent substitution of K_2_O by a single Na^+^ in NaOH), corresponding to a sodium hydroxide mass of 0.006052 mol × 40 g/mol = 0.24208 g. By adjusting the ratio, the alkalinity of the solution can be precisely controlled to ensure the optimization of material reaction activity.

#### 2.2.3. Mix Ratio Design

This experimental investigation systematically evaluated the influence of key mixture parameters on the mechanical properties and setting characteristics of ASRC through controlled variation in recycled stone powder content (AAS2-1 to AAS2-5), recycled rubber powder dosage (AAS3-1 to AAS3-6), binder-to-sand ratio (AAS4-1 to AAS4-3), and fiber content (AAS5-1 to AAS5-3). The study comprehensively assessed the effects of these parameters on compressive strength, flexural strength, and setting time. By integrating macro-scale performance testing with microstructural characterization techniques, this paper established an optimized mixture proportion for alkali-activated slag rubber concrete (ASRC) and elucidated the mechanical behavior, setting characteristics, and failure mechanisms of AASRM composites. The material combinations required per cubic meter of AASCMS are shown in Table 3.

**Figure 3:**
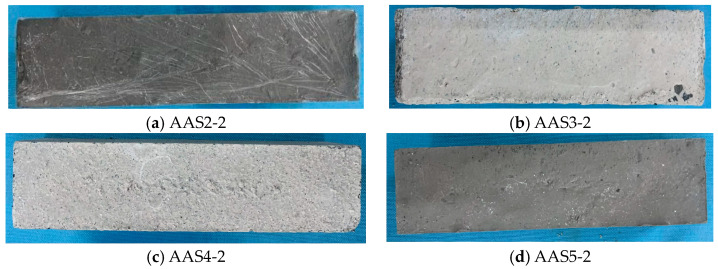
The finished product ASRCM test block.

Figure 3 presents representative specimens illustrating the curing outcomes and morphological features of the optimized ASRCM mixtures, demonstrating the material’s structural integrity and surface characteristics under different formulation conditions.

### 2.3. Testing Methods

#### 2.3.1. Compressive and Flexural Strength Tests

The prepared specimens were subjected to standardized curing regimes at intervals of 3, 7, and 28 days to evaluate age-dependent property development. Following each curing period, comprehensive mechanical characterization was performed, including flexural strength assessment through three-point bending tests and compressive strength measurement using axial loading, complemented by microstructural analysis through advanced characterization techniques.

**Figure 4:**
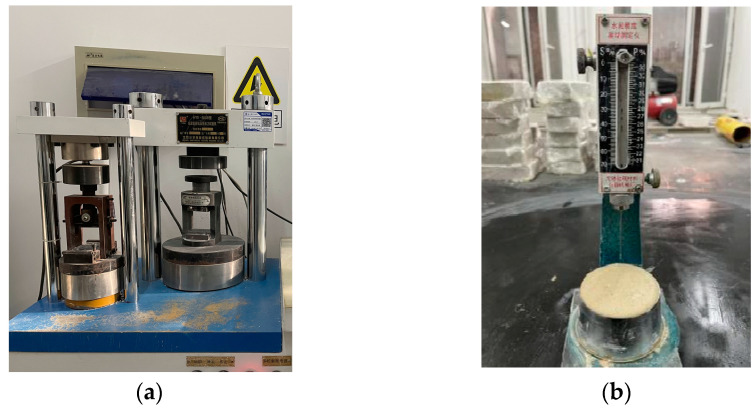
Testing equipment used: (**a**) compressive and flexural strength testing machine; (**b**) Vicat apparatus.

In accordance with method of testing cements—determination of strength (ISO method) (GB/T 17671-2021) [28], using the HYE-300B cement flexural and compressive constant stress testing machine, which manufactured by Quanzhou KeShuo Instrument Co., Ltd. (Quanzhou, China), as shown in Figure 4. The compressive strength of the intact specimens without any other damage was tested at a loading rate of 2400 N/s. The flexural strength of 40 × 40 × 160 mm mortar specimens was first tested. The flexural strength test results are determined by the arithmetic mean of six measured values obtained from three prism specimens. If any of the remaining five values deviates from the recalculated mean by more than ±10%, the test results shall be discarded and the test repeated. Individual compressive strength results are reported to the nearest 0.1 MPa, and the arithmetic mean is also rounded to 0.1 MPa.

Subsequently, compressive strength testing was executed on 40 × 40 × 160 mm ASRCM mortar specimens. The compressive strength test results were obtained following a procedure analogous to that of flexural strength testing.

#### 2.3.2. Setting Time Test

**Figure 5:**
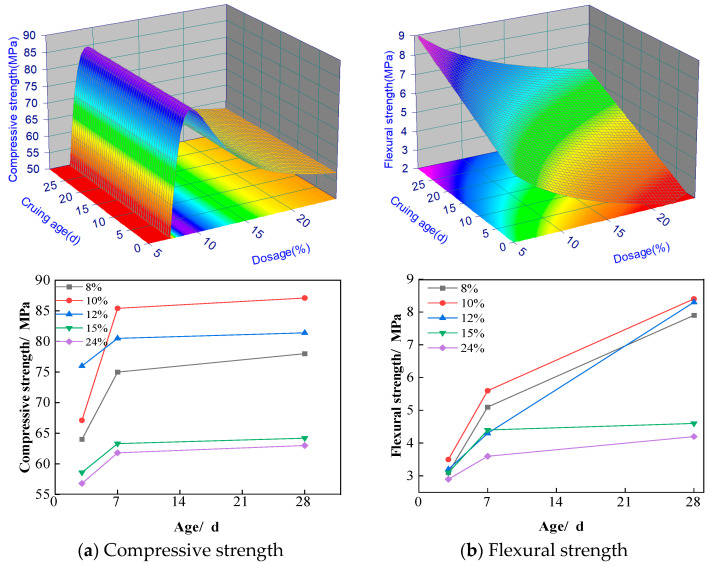
Effect of recycled stone powder on compressive and flexural resistance of materials.

To prepare the specimens for setting time tests, the well-mixed mortar was pulled into the round mold and placed in the curing chamber for 10 min. The specimens were then removed from the mold and tested using the Vicat apparatus, as shown in Figure 5b. The Vicat apparatus measured the initial setting time every 5 min. To obtain the setting time, the timer was started from the moment water was added and continued until the needle stopped sinking or, after releasing the needle for 30 s, the pointer reading was 4 mm ± 1 mm from the bottom plate. This time was measured as the initial setting time of the mortar.

Afterwards, the circular mold with an inner diameter of 65 mm was immediately rotated 180 degrees so that the top was facing downwards and then placed in the curing chamber. Measure every 15 min or less. The timing starts from the moment of adding water and continues until the final solidification needle approaches the sample and leaves no trace within 30 s of free fall. This is recorded as the final setting time.

#### 2.3.3. Microstructural Analysis Methods

Following gold products—analytical methods of scanning electron microscopy and energy-dispersive X-ray spectrometry (GB/T 17362-2008) [29], microstructural testing of ASRCM was performed:

(1) X-ray diffractometer (XRD): XRD tests were performed on the samples from the specimens using D8-Advance from Bruker Corporation (Billerica, MA, USA). Small specimens were pulverized into fine powder, passed through a 75 μm sieve to ensure sufficient surface area and minimize particle size effects, and dried to constant weight at 60 °C. The samples were scanned using an XRD instrument to identify mineral phases or crystalline structures.
(2) Scanning electron microscope (SEM): SEM analyses were performed on the specimens using SUPERTM 55 from Carl Zeiss AG (Oberkochen, Germany). Fiber-containing fragments from the central fractured region of ASRCM specimens were selected. After surface treatment, the fragments were gold coated under vacuum to ensure uniform conductive coverage. The coated samples were placed in the SEM, adjusted to appropriate working distances and accelerating voltages, and imaged at optimal resolutions to acquire high-quality micrographs.

## 3. Results and Discussions

### 3.1. Effect of Recycled Stone Powder on ASRCM Strength and Analysis

#### 3.1.1. Preferred Dosing of Recycled Stone Powder

The effects of regenerated stone powder doping on the compressive and flexural strengths of ASRCM are illustrated in Figure 5a,b. Figure 5a indicates that, with an increase in the amount of regenerated stone powder, the compressive flexural strength of the specimens first increases and then decreases. When the dosage is less than 12%, both the compressive and flexural strengths of the specimens increase significantly with the addition of more regenerated stone powder. After 28 days of curing, although there is no significant change in compressive strength, the flexural strength shows a marked increase compared to other curing ages. However, when the dosage is higher than 15% of regenerated stone powder, both compressive and flexural strengths were not shown to significantly increase, and there may even be a tendency for them to decrease. When comparing strengths from 7 to 28 days, especially for 8–12% of regenerated stone powder, a significant increase in flexural strength is shown, while there is no significant increase in compressive strength from 7 to 28 days. A few factors might contribute to this phenomenon, including:

(1) The addition of recycled stone powder improves the pore structure of the material, making it more homogeneous and denser. This improvement had a significant effect on the flexural strength of the material, which is closely related to the homogeneity and continuity of the material. At 28 days, further optimization of the pore structure significantly improved the flexural strength. The recycled stone powder may also have formed a more stable bond at the interfacial transition zone between the cement matrix and the aggregate, thus improving the overall toughness and flexural properties of the material. This interfacial enhancement is more significant for flexural strength, while the effect on compressive strength is relatively small.

(2) The hydration reaction of the matrix has been more sufficient at 7 days, and most of the active ingredients have been involved in the hydration reaction, forming stable hydration products. This makes the compressive strength already reach a high level at an early stage. From 7 to 28 days, the rate of hydration reaction gradually slows down, and the additional hydration products have limited contribution to the strength. As a result, the compressive strength did not increase significantly at the later stage, whereas the flexural strength increased significantly due to further optimization of the pore structure. 

(3) The flexural strength is more sensitive to the microstructure and pore distribution of the material. The addition of recycled stone powder may have improved the toughness and crack resistance of the material at the micro level, which significantly increased the flexural strength. The compressive strength, on the other hand, mainly reflects the overall strength of the material and is relatively unresponsive to improvements in microstructure. Therefore, even if the pore structure is optimized, the increase in compressive strength is not as significant as the flexural strength.

Therefore, after comprehensive consideration, we have chosen a doping level of 10% regenerated stone powder and a curing age of 28 days. At this point, the flexural strength is 18% higher than that of ordinary M30 grade cement mortar, while the compressive strength is 15% higher than that of ordinary M30 grade cement mortar, indicating a more pronounced enhancement in strength. Based on the experimental dataset, a second-order response surface mathematical model was developed to characterize the material behavior, with the statistically significant predictive equations formulated as follows.

(1) $$\begin{array}{c} F_{c1}=e^{3.97+1413.2e^{-x}+51.49/x^{2}}\\ F_{f1}=4.89-0.86lnx+0.085y \end{array}$$

where $F_{c1}$ is the compressive strength, MPa; $F_{f1}$ is the flexural strength, MPa; *x*(0–30) is the curing age, d; and *y*(0–25) is the dosage of the recycled stone powder, %.

#### 3.1.2. Recycled Stone Powder Mechanism

To accurately regulate the action pathways of recycled stone powder in ASRCM and provide theoretical support for the development of high-volume, high-performance solid waste-based cementitious materials, this study also analyzes the action mechanism of recycled stone powder. The mechanism by which regenerated stone powder enhances ASRCM involves the provision of additional MgO, CaO, and SiO_2_. The hydration of MgO in aqueous media generates Mg^2+^ ions and OH^−^, establishing an alkaline environment (pH 9–12) conducive to structural assembly. Subsequently, SO_4_^2−^ are intercalated into the interlayer spaces of the evolving magnesium-aluminum layered double hydroxide (LDH) framework through electrostatic interactions, effectively neutralizing the positively charged brucite-like layers. It leads to the formation of hydrotalcite (Ht), calcium hydrate sulfate, and the generation of OH^−^ ions, which will be analyzed using the XRD analysis in Section 3.6. The high concentration of OH^−^ penetrates the interior of the slag vitreous body, disrupting its network structure and accelerating the dissolution of the slag vitreous body. Additionally, the finer particle size of regenerated stone powder results in a larger specific surface area and an increased surface area in contact with the alkali excitation solution, thereby accelerating the hydration reaction of the slag. This rapid hydration is more likely to enhance the bond between ASRCM and the cured material, making it suitable for multiple pours or repair applications. The fine powder particles fill the voids in the slag, and this pore filling contributes to a denser ASRCM, significantly improving its flexural strength as demonstrated in Figure 5.

However, the fine surface area of additives does have an impact on the construction properties. If the specific surface area is too high, it may lead to uneven dispersion of the additives in the concrete, affecting their compatibility with the cement and hence the working properties of the concrete. Therefore, the particle size of recycled stone powder selected in this study was 6.6 μm, which is smaller in size, in order to ensure its uniform dispersion in concrete during the construction process, while at the same time improving the construction performance and final quality of concrete. This analysis aims to establish a scientific foundation for optimizing the utilization of recycled stone powder, which not only enhances the understanding of its interaction with other components (e.g., recycled rubber and flax fiber) in the composite system but also facilitates the rational design of sustainable construction materials.

### 3.2. Effect of Recycled Rubber on ASRCM Strength and Analysis

#### 3.2.1. Preferred Dosing of Recycled Rubber

**Figure 6:**
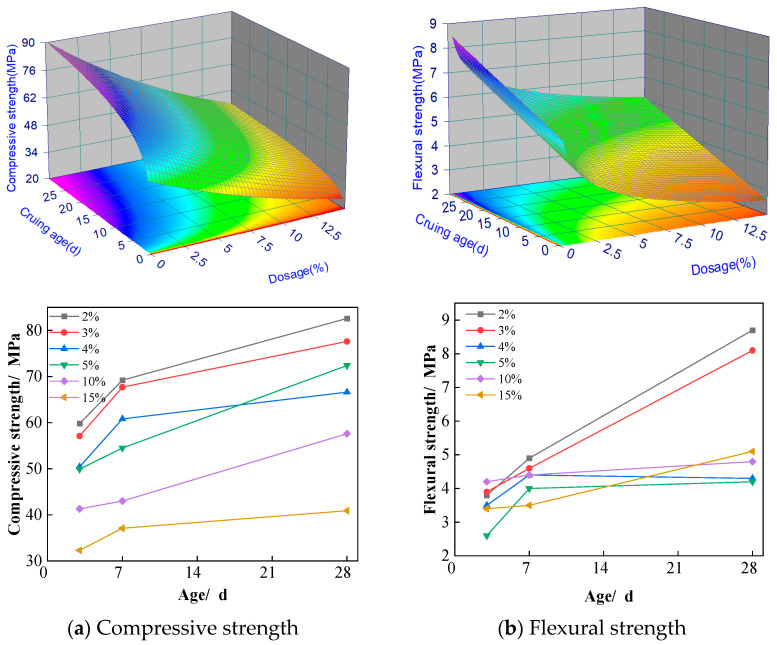
Effect of recycled rubber on compressive and flexural resistance of materials.

The effect of recycled rubber powder dosage on the compressive and flexural strengths of ASRCM is illustrated in Figure 6a,b. As the dosage of recycled rubber powder increases, the compressive strength of the specimens consistently decreases for both 7 days and 28 days. Notably, the enhancement of compressive strength was most pronounced at dosages of 2% and 3%. Also, from 2% to 3%, the flexural strength was similar and remained very high. However, as the dosage of the rubber powder increased above 4%, the flexural strength significantly decreased for both 7 days and 28 days, and the flexural strength did not show a significant gain from 7 days to 28 days. A few factors might have contributed to this phenomenon, including:

(1) When there is a small dosage of rubber powder of 2% to 3% in the mix, it disperses better in the material, and these particles are effective in filling the pores in the material and improving the uniformity and densification of the pore structure. This optimization significantly improved the toughness and crack resistance of the material and significantly increased the flexural strength between 7 and 28 days. In contrast, when the rubber powder is doped at a level greater than 5%, the rubber particles back in the material undergo agglomeration and form larger clusters, and these clusters disrupt the homogeneity of the material and increase the number of defects and stress concentration points within the material. Therefore, although the rubber powder itself has good toughness and ductility, an excessive amount of rubber powder will instead reduce the overall performance of the material, resulting in no significant change in bending strength.

(2) The small dosing of rubber powder at 2% to 3% may help to form a good interfacial bond between the rubber particles and the cement matrix. This bond improves both the toughness and ductility of the material and enhances the crack resistance of the material, thus significantly increasing the flexural strength. When the dosage of rubber powder is greater than 5%, the agglomeration of rubber particles may lead to a decrease in the interfacial bond strength. The interface between the rubber particles and the matrix may become weak, leading to uneven stress transfer, which in turn affects the overall performance of the material, resulting in no significant change in bending strength.

(3) 2% to 3% rubber powder dosing may have less effect on the hydration reaction, which can continue between 7 and 28 days to further optimize the microstructure of the material, thus improving the bending strength. When the dosage is greater than 5%, it will have a greater effect on the hydration reaction, preventing the hydration reaction from proceeding further, resulting in no significant improvement in the microstructure of the material between 7 and 28 days, and therefore no significant change in the bending strength.

In addition, considering that recycled rubber improves the brittleness and frost resistance of ASRCM specimens, and adhering to the principle of maximizing the utilization of bulk solid waste, a small dosage of recycled rubber will be more appropriate. Thus, a dosage of 3% recycled rubber was selected to be the optimized dosage. At a curing age of 28 days, the flexural strength is 8% higher than that of ordinary M30 grade cement mortar, and the compressive strength is 6% higher than that of ordinary M30 grade cement mortar. Additionally, the toughness of the specimens was significantly enhanced during the flexural test. Among all test groups, the results were more satisfactory, particularly when combined with the maintenance age of specimens mixed with recycled stone powder. Therefore, 28 days was ultimately chosen as the maintenance age. Based on the experimental dataset, a second-order response surface mathematical model was developed to characterize the material behavior, with the statistically significant predictive equations formulated as follows.

(2) $$\begin{array}{c} F_{c2}=e^{4.06+0.05x+0.13lny}\\ F_{f2}=4.89+0.86lnx+0.09y \end{array}$$

where $F_{c2}$ is the compressive strength, MPa; $F_{f2}$ is the flexural strength, MPa; *x*(0–30) is the curing age, d; and *y*(0–13) is the dosage of the recycled rubber, %.

#### 3.2.2. Recycled Rubber Mechanism of Action

The mechanism by which recycled rubber enhances ASRCM can be attributed to three primary aspects. Firstly, the rubber particles effectively fill the voids within the slag matrix, thereby enhancing the overall stability of ASRCM. This void-filling action reduces water penetration and associated damage, consequently improving the material’s durability and crack resistance, which is shown in Figure 6 for enhancing the flexural strength with a small dosage of rubber powder. Additionally, the inherent elasticity and toughness of rubber powder enable it to absorb impact energy efficiently, significantly enhancing the concrete’s impact resistance. Under impact loading, these rubber particles mitigate the progression from initial cracking to complete failure, alleviate stress concentration at crack tips, and reduce the brittle failure typically associated with high-strength ASRCM, thereby improving the material’s ductility. Secondly, in the alkaline environment of ASRCM, the surface of rubber particles undergoes oxidation and degradation, resulting in a roughened texture that forms a strong bond with the ASRCM matrix. The alkaline environment of ASRC, through the dual action of chemical oxidation and physical etching, results in the formation of an active rough interface on the surface of the rubber particles, which not only enhances the bonding force through mechanical interlocking but also optimizes the interfacial properties through chemical bonding. Thus, it improves the interfacial bond strength and overall mechanical stability of the composite material to an approximate 5% increase in the concrete’s compressive strength. Finally, the incorporation of recycled rubber particles modifies the pore structure of the ASRCM matrix, leading to reduced porosity and consequently enhancing the material’s durability. Similar to recycled stone powder, this analysis aims to reveal the interaction mechanisms between recycled rubber and cementitious systems, thereby filling the research gap in the role of recycled rubber in cementitious materials and providing theoretical support for the rational design of high-performance cementitious materials.

### 3.3. Effect of Flax Fiber on ASRCM Strength and Analysis

#### 3.3.1. Preferred Dosing of Flax Fiber

**Figure 7:**
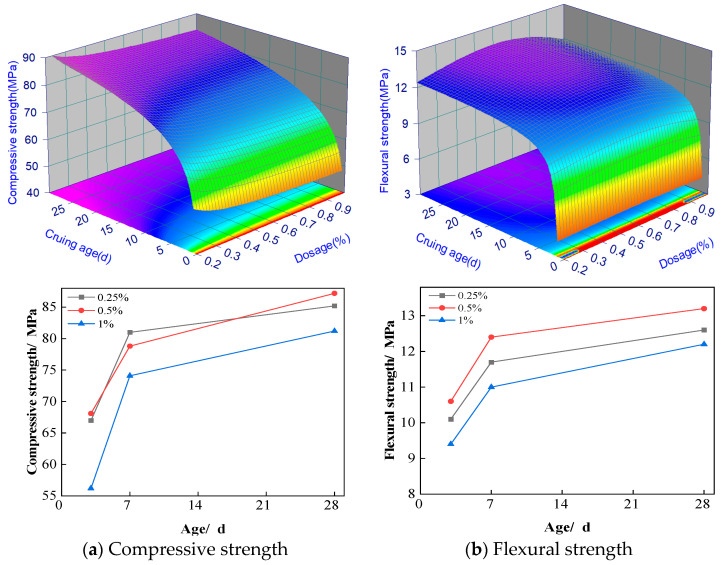
Effect of flax fiber on compressive and flexural resistance of materials.

The effects of flax fiber dosage on the compressive and flexural strengths of ASRCM are illustrated in Figure 7a,b. The experimental results demonstrate that at 7 days, the compressive strength decreased with fiber dosage increasing from 0.2% to 1%. While at 28 days, it increases with fiber dosage increasing from 0.2% to 0.5% but decreases with further increasing fiber dosage from 0.5% to 1%, which is similar to the flexural strength at both 7 days and 28 days. The comparative analysis of different flax fiber dosages clearly reveals that the optimal enhancement occurs at a dosage of 0.5%. Specifically, at 28 days of curing, this fiber dosage compared to ordinary M30 cement mortar has increased its compressive strength by 20%, and its flexural strength has increased by 68%, indicating an excellent mechanical property. These findings suggest that the incorporation of flax fibers at appropriate dosages can effectively improve the mechanical properties of ASRCM, particularly during the early curing stages. Based on the experimental dataset, a second-order response surface mathematical model was developed to characterize the material behavior, with the statistically significant predictive equations formulated as follows.

(3) $$\begin{array}{c} F_{c3}=50.58+1.875/x+lnyF_{f2}=4.89+0.86lnx+0.09y\\ F_{f3}=\frac{1}{0.07+0.05x^{2}lnx+0.03/lny} \end{array}$$

where $F_{c3}$ is the compressive strength, MPa; $F_{f3}$ is the flexural strength, MPa; *x*(0–30) is the curing age, d; and *y*(0–1) is the dosage of the recycled rubber, %.

#### 3.3.2. Flax Fiber Mechanism of Action

The reinforcing mechanism of flax fiber in ASRCM can be attributed to four primary aspects. Firstly, the inherent tensile strength and toughness of flax fibers, combined with their natural spindle structure and unique pectinic beveled-edge pore morphology, enable effective bridging between slag particles. This structural characteristic enhances interfacial bonding and interaction forces, thereby improving the mechanical strength and stability of the composite material, as shown in Figure 7. Secondly, the incorporation of flax fibers significantly modifies the thermal properties of ASRCM, particularly by reducing thermal conductivity and enhancing thermal resistance, which contributes to improved material stability and energy efficiency under varying environmental conditions. The improvement of the thermal properties of ASRC by flax fibers and compressive strength is a synergistic effect between the interface optimization, structural enhancement, thermal stress buffer, and other multi-scale mechanisms. Thus, to maintain the mechanical properties, such as compressive strength higher than 45 MPa, the thermal conductivity could be reduced to below 0.7 W/(m·K), to meet the needs of the building envelope on the mechanical-insulation integration performance. So, if the composite is to be used under high-temperature conditions, the basic mechanical properties of the components can be guaranteed with reduced thermal conductivity. Thirdly, flax fibers play a crucial role in modifying the internal microstructure of concrete, effectively reducing the formation and propagation of both plastic shrinkage cracks and internal microcracks, while enhancing the continuity of the concrete matrix, ultimately leading to superior overall performance. Finally, the unique properties of flax fibers, including their resistance to freeze–thaw cycles, chloride ion penetration, water permeability, and carbonation, contribute to the densification of ASRCM. This microstructural refinement results in reduced permeability and significantly enhanced durability of the concrete composite. This analysis aims to elucidate the enhancement or degradation mechanisms through which flax fibers affect the strength, toughness, and long-term service performance of ASRCM. The insights gained here are critical for establishing a theoretical basis for the targeted optimization of material properties, enabling the rational design of flax fiber-reinforced ASRCM with balanced performance attributes. Specifically, this research addresses the knowledge gap in understanding fiber-induced improvements or drawbacks, thereby facilitating the development of sustainable, high-performance construction materials that leverage natural fibers to enhance engineering performance while promoting ecological sustainability.

### 3.4. Preferred Sand Ratio

**Figure 8:**
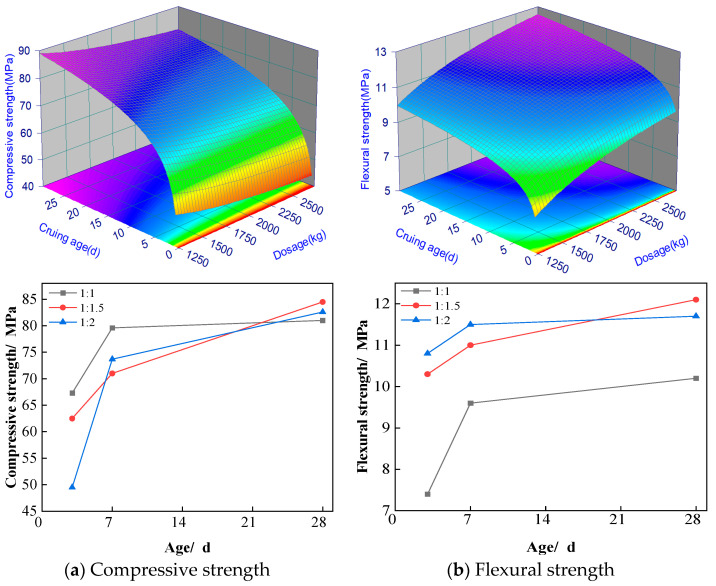
Effect of sand ratio on compressive and flexural resistance of materials.

The influences of the rubber-to-sand ratio on the compressive and flexural strengths of ASRCM are presented in Figure 8a,b. The experimental results demonstrate a non-linear relationship between the rubber-to-sand ratio and mechanical strength, characterized by an initial increase followed by a subsequent decrease. While specimens with a preliminary rubber-to-sand ratio of 1:1.5 exhibited slightly inferior enhancement effects compared to those with a ratio of 1:1.2 during early curing stages, a significant improvement was observed at 28 days of curing. Specifically, the 1:1.5 ratio specimens demonstrated superior mechanical performance; their flexural strength is 20% higher than that of ordinary M30 cement mortar, and their compressive strength is 15% higher. It is also noted from Figure 8a that at a rubber-to-sand ratio of 1:1, there is no significant increase in compressive strength from 7 to 28 days. The stabilization of compressive strength for a rubber-to-sand ratio of 1:1 from 7 to 28 days might have been brought about by a few factors, including:

(1) the saturation of hydration reaction. At 7 days the hydration reaction has been more sufficient; most of the active ingredients have been involved in the hydration reaction, forming stable hydration products. With the passage of time to 28 days, the rate of hydration reaction gradually slowed down, and the additional hydration products had limited contribution to the strength;

(2) the stabilization of the pore structure. At 7 days, the pore structure of the material may have been basically stabilized, and the subsequent hydration reaction has less effect on the improvement of the pore structure. The stabilization of the pore structure means that it is difficult to significantly improve the compactness and strength of the material at a later stage;

(3) the early strength of ASRCM develops rapidly. In the early stage (7 days), the strength of the material develops rapidly and may have reached a high level. This rapid strength development may have consumed most of the active ingredient, resulting in slow or stagnant strength growth in the later stages; and

(4) the experimental environmental conditions (e.g., temperature, humidity) may have an effect on late strength development. Low humidity in the experimental environment leads to water loss from the surface of the material, which affects the continuation of the hydration reaction, thus limiting further strength increase.

These findings suggest that the optimal rubber-to-sand ratio for maximizing long-term mechanical properties in ASRCM lies within this range, highlighting the importance of proper proportioning in achieving optimal performance characteristics. Based on the experimental dataset, a second-order response surface mathematical model was developed to characterize the material behavior, with the statistically significant predictive equations formulated as follows.

(4) $$\begin{array}{c} F_{c4}=41.82+17820/x+9.7lny\\ F_{f4}=12.09-5940/x+0.77lny \end{array}$$

where $F_{c4}$ is the compressive strength, MPa; $F_{f4}$ is the flexural strength, MPa; *x*(0–30) is the curing age, d; and *y*(1250–2500) is the dosage of the recycled rubber, %.

### 3.5. Preferred Resting Time of Alkali Excitation Solution

**Figure 9:**
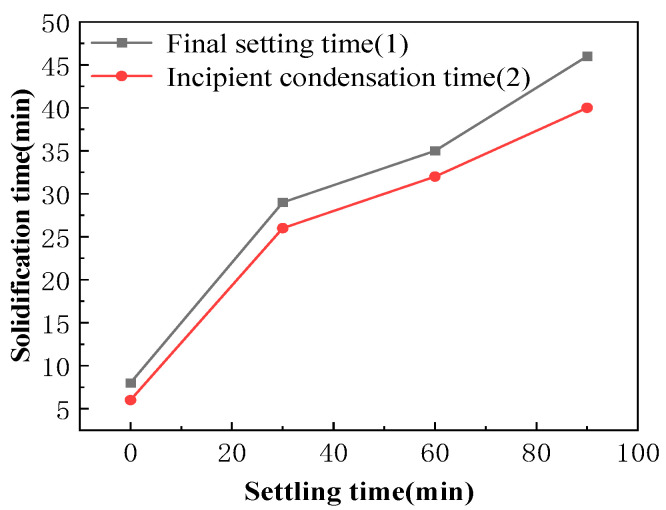
The effect of heat release time on the setting time of ASRCM.

The effect of alkali activator solution resting time on the setting characteristics of ASRCM is illustrated in Figure 9. Experimental results reveal that the initial and final setting times of ASRCM are closely correlated, with a consistent time difference maintained within 10 min. The study demonstrates a direct relationship between the resting time of the alkali activator solution and the setting time of ASRCM, showing a gradual prolongation of setting time with increased solution resting duration. When the solution exhibits maximum exothermic activity at 40 ± 5 °C, the ASRCM demonstrates rapid setting behavior with a final setting time of only 7–9 min. Conversely, when the resting time extends to 90 min, allowing the solution temperature to equilibrate to ambient conditions (20 ± 5 °C), the final setting time significantly increases to 40–45 min, achieving the desired retardation effect. This phenomenon can be attributed to the thermal dynamics of the activation process: prolonged resting time allows the solution temperature to approach ambient conditions, thereby reducing the kinetic energy of ionic species and subsequently slowing down the hydration reaction. This temperature-dependent retardation effect decreases the decomposition rate of vitreous slag components and magnesium oxide while simultaneously moderating the formation kinetics of hydration products, including C-S-H gel, Ht, and M-A-S-H gel. Consequently, the extended resting time, through its temperature-reducing effect, effectively prolongs the setting time of ASRCM by modulating the reaction kinetics of the alkali activation process.

### 3.6. Microscopic Analysis

#### 3.6.1. X-Ray Diffraction (XRD) Analysis

**Figure 10:**
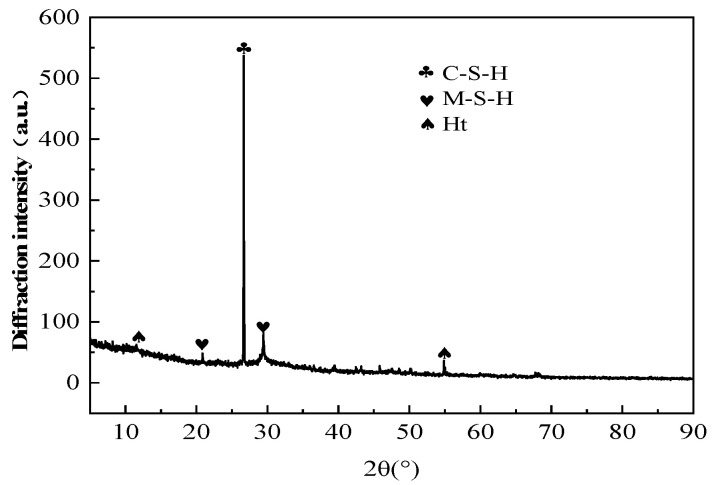
The XRD analysis of the preferred ratio.

The XRD analysis of the optimized mixture ratios, as presented in Figure 10. The preferred formulation consisted of recycled stone powder and recycled rubber at 10% and 3% of slag mass, respectively, with a binder-to-sand ratio of 1:1.5, a fiber content at 0.5% of slag mass, and a water glass modulus of 1.0. Figure 10 reveals four distinct characteristic peaks that enable the identification of specific phase components through their corresponding diffraction angles. Phase analysis indicates that the primary hydration products in the ASRCM system consist of calcium silicate hydrate (CSH) gel and magnesium aluminosilicate hydrate (M-A-S-H) gel, accompanied by minor quantities of hydrotalcite (Ht). It was learned from reviewing the relevant literature that the recycled stone powder does not usually show up as a separate phase in XRD analysis. This is because recycled stone powder is mainly composed of silicate minerals with similar composition and structure to natural silicate minerals, such as quartz (SiO_2_). In the XRD pattern, the characteristic peaks of recycled stone powder may overlap with those of minerals such as quartz. Therefore, in XRD analysis, the peaks of recycled stone powder may not be recognized as a separate phase but contribute to the XRD pattern together with other mineral components in the matrix. CSH is an amorphous or semi-crystalline hydration product; theoretically, it usually does not show a very sharp peak in the XRD pattern, and its characteristic peak may appear at around 30°. However, in practice, the shape and intensity of the CSH peak may vary depending on the specific composition of the sample and preparation conditions. In Figure 10, the peak of CSH showed around 26° while appearing to be sharp, indicating that the intensity of the CSH peak might have been influenced by a number of factors, such as the specific preparation conditions of the experimental samples, the high sensitivity and resolution of the XRD instrument, and the presence of other phases in the samples that have an effect on the peak shape.

The diffraction intensity analysis demonstrates a gradual decrease in peak intensity with increasing temperature; however, this reduction is relatively insignificant due to the remarkable thermal stability of the hydration products and mineral phases formed at the optimized mixture ratios. This thermal stability can be attributed to the well-balanced chemical composition and stable crystalline structures achieved through the optimized formulation. This finding confirms that the incorporation of recycled regenerative materials makes ASRC maintain a more stable state even under high-temperature conditions, which is of great importance to the strength of the building in high-temperature applications.

#### 3.6.2. SEM Analysis of Recycled Stone Powder Reinforced ASRCM

**Figure 11:**
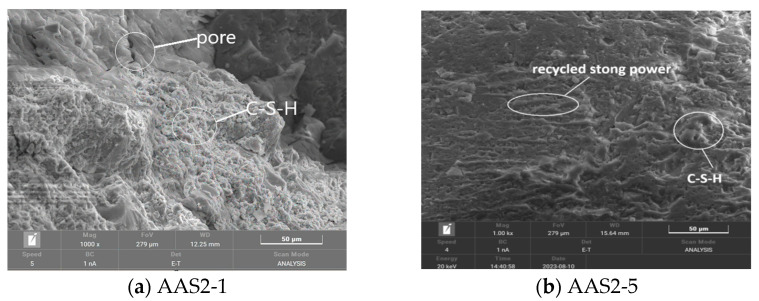
The phase of ASRCM crystal.

Figure 11a,b shows the SEM images of the ASRCM reinforced by recycled stone powder. Although the location of CSH and MSH cannot be pinpointed by SEM scanning, the extent of their presence can be determined. In this study, the SEM images from previous research [14,15,16] showed that CSH typically presents an irregular gel-like structure, MSH usually shows a more regular crystal morphology, while the regenerated stone powder would present an irregular granular morphology, and the particles are aggregated in the matrix and present an irregular texture. By examining these morphological features, the locations of CSH, MSH, and recycled stone powder were preliminarily determined, and examples of these locations were labeled in the SEM images. In addition, Figure 11a also shows that certain voids exist in C-S-H gel. As regenerated stone powder is distributed in granular form between the C-S-H gel, alkali exciter can overcome the slag excitation activation energy, destroy the slag silica–oxygen network structural layer, and form the C-S-H gel, leading to a decrease in the concentration of Ca^2+^ ions. The reduction in Ca^2+^ ion concentration can further accelerate the diffusion of OH^−^ ions to the inside of the slag and significantly reduce the Ca(OH)_2_ crystals until all of them are consumed, resulting in a denser hardened ASRCM. At the same time, MgO can partially react with SiO_2_ and Al_2_O_3_ in the slag to form an M-A-S-H gel to provide strength, and on the other hand, partially form Ht in the alkaline environment, which distributes between the C-S-H gels to limit the drying shrinkage of the ASRCM through the volumetric expansion of the hydration process.

Comparative analysis of Figure 11b reveals that while the majority of C-S-H gels exhibit a dense microstructure, residual porosity persists within the matrix. In contrast to conventional alkali-activated slag cementitious materials (AASCM), the incorporation of recycled stone powder introduces additional Ca^2+^, Mg^2+^, and SiO_4_^4−^ ions, which significantly enhance the formation of amorphous hydration products, including C-S-H gels, Ht, and M-A-S-H gels. Notably, the system is devoid of thermally unstable crystalline phases typically found in ordinary Portland cement systems, such as calcium aluminate and calcium hydroxide, which are prone to decomposition at elevated temperatures. Furthermore, the unreacted fraction of recycled stone powder, owing to its finer particle size distribution compared to slag, effectively fills the interstitial pores, thereby contributing to microstructural densification. This unique combination of enhanced hydration product formation and pore-filling effect imparts superior binding properties to the system, resulting in improved mechanical performance and durability characteristics.

**Figure 12:**
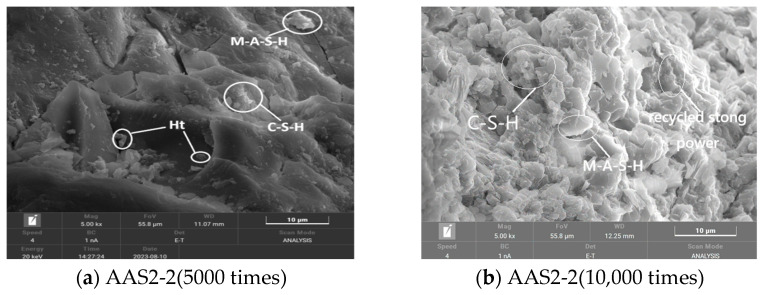
The SEM diagram of ASRCM reinforced by recycled stone powder.

The influence of recycled stone powder dosage on ASRCM strength is primarily mediated through its effect on the degree of surface densification of the colloidal matrix. As demonstrated in Figure 12a, an insufficient dosage of recycled stone powder fails to achieve effective void filling, leaving residual porosity within the matrix and consequently providing limited strength enhancement. Conversely, Figure 12b illustrates that excessive dosage results in incomplete reaction of the stone powder, leading to particle agglomeration and free dispersion within the matrix. This over-saturation condition not only prevents complete pore filling but also creates weak interfaces that compromise the mechanical integrity of the composite. Optimal performance is achieved at moderate dosages, where the recycled stone powder effectively enhances matrix densification through two mechanisms: (1) participation in the hydration reaction to form additional binding phases and (2) physical filling of interstitial pores due to its fine particle size distribution. This dual functionality of moderate stone powder addition results in improved microstructural homogeneity and enhanced mechanical properties of the ASRCM composite.

#### 3.6.3. SEM Analysis of Recycled Rubber Reinforced ASRCM

**Figure 13:**
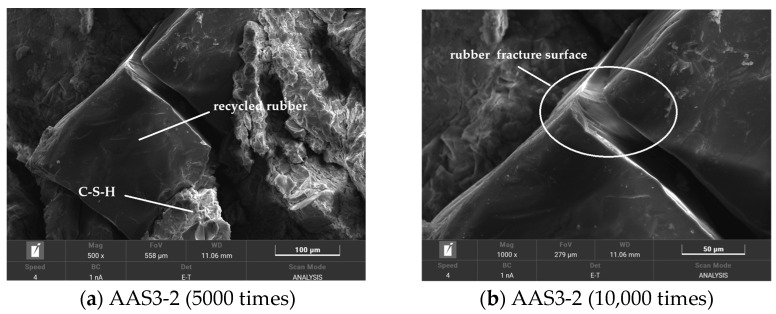
The bonding surface between recycled rubber and matrix.

Microstructural analysis presented in Figure 13 reveals the interfacial characteristics between recycled rubber particles and the ASRCM matrix. The bonding strength at the rubber–matrix interface significantly exceeds the intrinsic strength of the rubber particles themselves, as evidenced by the observation that matrix–rubber adhesion persists even when rubber particles undergo fracture. This strong interfacial bonding enables effective stress transfer and demonstrates excellent matrix–rubber compatibility. The incorporation of recycled rubber, even at low concentrations, substantially enhances the material’s elastic properties and significantly improves the composite’s ductility. However, excessive rubber content leads to particle agglomeration and surface adsorption on the matrix, resulting in disproportionate increases in elasticity and consequent reductions in both flexural and compressive strength. Therefore, optimal performance requires precise control of recycled rubber content to achieve the desired balance between enhanced ductility and maintained mechanical strength, ensuring the structural integrity and performance characteristics of the ASRCM composite for enhanced mechanical properties of the ASRCM composite.

#### 3.6.4. SEM Analysis of Flax Fiber-Reinforced ASRCM

**Figure 14:**
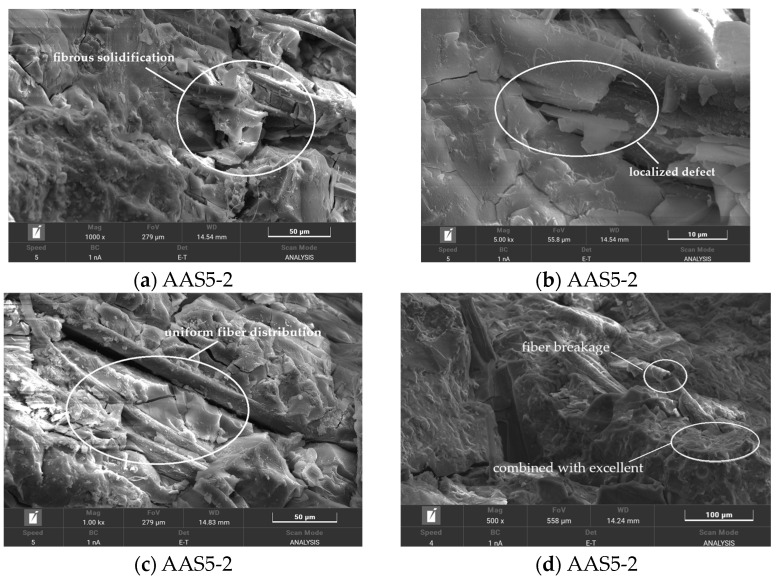
The SEM diagram of flax fiber-reinforced ASRCM.

Microstructural analysis presented in Figure 14a,b demonstrates that untreated fibers exhibit poor dispersion within the ASRCM matrix, forming characteristic flocculent structures. This inadequate dispersion results in fiber entanglement and cluster formation within the ASRCM slurry, creating localized defects that compromise both structural homogeneity and mechanical performance. The heterogeneous fiber distribution induces stress concentration points and facilitates crack propagation, thereby negatively impacting the material’s load-bearing capacity and long-term durability. In contrast, Figure 14c,d reveal significant morphological improvements in pretreated fibers, showing effective dispersion of fiber bundles into discrete, individual units. The pretreatment process yields a more homogeneous fiber distribution within the ASRCM slurry, substantially reducing fiber entanglement and optimizing the composite’s microstructure. This enhanced dispersion state contributes to improved matrix compactness and uniform stress distribution, ultimately leading to superior mechanical properties, including enhanced strength and toughness characteristics of the ASRCM composite.

## 4. Conclusions

This paper systematically evaluated the influence of key mixture parameters on the mechanical properties and setting characteristics of ASRC through controlled variation in recycled stone powder content, recycled rubber powder dosage, binder-to-sand ratio, and fiber content. The key findings of this study can be summarized as below:

(1) The preferred formulation consists of recycled stone powder and recycled rubber at 10% and 3% of slag mass, respectively, with a binder-to-sand ratio of 1:1.5. Fiber content was optimized at 0.5% of slag mass, and the water glass modulus was maintained at 1.0. This optimized composition represents a balanced integration of waste materials and performance-enhancing additives, demonstrating the potential for sustainable construction material development while maintaining superior mechanical characteristics.

(2) The experimental results demonstrate a non-linear relationship between recycled material content and mechanical properties. The incorporation of recycled stone powder exhibits a characteristic parabolic effect on strength development, with optimal performance achieved at 10% dosage, resulting in 18% and 15% enhancements in flexural and compressive strength, respectively. While increased recycled rubber content generally reduces compressive and flexural strength, its incorporation at 3% significantly improves the material’s ductility and frost resistance, with minimal strength reduction and substantial toughness enhancement during flexural testing. Fiber reinforcement demonstrates remarkable effectiveness at 0.5% dosage, yielding 68% and 20% improvements in flexural and compressive strength, respectively. The optimal rubber-to-sand ratio of 1:1.5 produces maximum mechanical performance, with 20% and 15% increases in flexural and compressive strength. Furthermore, extending the alkaline activator solution resting time to 90 min effectively prolongs the final setting time to 40–45 min, achieving satisfactory retardation effects while maintaining material integrity.

(3) Microstructural characterization through XRD and SEM analyses reveals that the primary hydration products in ASRC consist of calcium silicate hydrate (C-S-H) gel, magnesium aluminosilicate hydrate (M-A-S-H) gel, and minor quantities of hydrotalcite (Ht). The recycled stone powder particles are uniformly distributed within the pore structure, effectively enhancing matrix densification. Hydrotalcite formation within the C-S-H gel matrix plays a crucial role in mitigating drying shrinkage through controlled volumetric expansion during hydration. The interfacial bonding strength between rubber particles and the cementitious matrix significantly exceeds the intrinsic strength of rubber itself, providing substantial elastic properties and enhanced ductility to the composite material. Furthermore, flax fibers establish strong interfacial bonding with the matrix, creating an effective reinforcement network that substantially improves the overall mechanical strength and structural integrity of the ASRC material.

The integration of recycled materials into ASRCM research has established novel pathways for construction waste recycling. The application of recycled materials in construction demonstrates substantial economic merits through three synergistic mechanisms: Primarily, it significantly reduces raw material procurement costs and waste disposal expenditures while mitigating dependence on natural resources and enhancing resource utilization efficiency. Secondarily, enterprises utilizing construction-derived recycled building materials processed from industrial byproducts and demolition waste qualify for governmental tax incentives, subsidies, and green building certification credits, thereby reinforcing market competitiveness. This strategic positioning facilitates market penetration into green construction sectors, generating long-term economic returns while advancing circular economy principles through closed-loop material cycles. However, as this study is limited to the lab testing, future further investigations on the practical applications of the ASRC materials and their field implementations are of interest.

## Figures and Tables

**Table 1:** Chemical composition of slag.

Component	SiO_2_	Al_2_O_3_	CaO	MgO	Fe_2_O_3_	SO_2_	Others	Loss on Ignition
Content (%)	36.90	15.66	37.57	9.30	0.36	-	0.57	-

**Table 2:** The Chemical composition of recycled stone powder.

Component	SiO_2_	AI_2_O_3_	CaO	MgO
Content (%)	36.92	9.58	26.85	14.64

**Table 3:** Mix ratio design.

Number	Slag (kg)	Recycled Stone Powder (kg)	Recycled Rubber (kg)	Sand (kg)	Flax Fiber (%)	Solution Standing Time (min)
AAS1	1200.00	/	/	/	/	/
AAS2-1	1200.00	100.00 (8%)	/	/	/	/
AAS2-2	1200.00	120.00 (10%)	/	/	/	/
AAS2-3	1200.00	140.00 (12%)	/	/	/	/
AAS2-4	1200.00	180.00 (15%)	/	/	/	/
AAS2-5	1200.00	280.00 (24%)	/	/	/	/
AAS3-1	1200.00	/	24.00 (2%)	/	/	/
AAS3-2	1200.00	/	36.00 (3%)	/	/	/
AAS3-3	1200.00	/	48.00 (4%)	/	/	/
AAS3-4	1200.00	/	60.00 (5%)	/	/	/
AAS3-5	1200.00	/	120.00 (10%)	/	/	/
AAS3-6	1200.00	/	180.00 (15%)	/	/	/
AAS4-1	1200.00	120.00 (10%)	36.00 (3%)	1320.00	/	/
AAS4-2	1200.00	120.00 (10%)	36.00 (3%)	1980.00	/	/
AAS4-3	1200.00	120.00 (10%)	36.00 (3%)	2640.00	/	/
AAS5-1	1200.00	120.00 (10%)	36.00 (3%)	1980.00	0.25	/
AAS5-2	1200.00	120.00 (10%)	36.00 (3%)	1980.00	0.50	/
AAS5-3	1200.00	120.00 (10%)	36.00 (3%)	1980	1.00	/
AAS6-1	1200.00	120.00 (10%)	36.00 (3%)	1980.00	0.50	30
AAS6-2	1200.00	120.00 (10%)	36.00 (3%)	1980.00	0.50	60
AAS6-3	1200.00	120.00 (10%)	36.00 (3%)	1980.00	0.50	90

## Data Availability

The original contributions presented in this study are included in the article. Further inquiries can be directed to the corresponding authors.
